# Advances in *Leishmania* Research: From Basic Parasite Biology to Disease Control

**DOI:** 10.3390/microorganisms11030696

**Published:** 2023-03-08

**Authors:** Nuno Santarém, Luís Cardoso, Anabela Cordeiro-da-Silva

**Affiliations:** 1 Parasite Disease Group, Institute for Research and Innovation in Health (i3S), University of Porto, 4200-135 Porto, Portugal; 2 Department of Biological Sciences, Microbiology Laboratory, Faculty of Pharmacy, University of Porto, 4050-313 Porto, Portugal; 3 Department of Veterinary Sciences, and CECAV—Animal and Veterinary Research Centre, University of Trás-os-Montes e Alto Douro (UTAD), 5000-801 Vila Real, Portugal; 4 Associate Laboratory for Animal and Veterinary Sciences (AL4AnimalS), 5000-801 Vila Real, Portugal

The genus *Leishmania* (Trypanosomatida: Trypanosomatidae) currently comprises just over 50 species, of which about 20 cause several syndromes in humans, collectively known as leishmaniasis or “leishmaniases”. The plural term emphasizes not a single and simple nosological entity, but rather a group of conditions resulting from distinct etiological agents and a wide spectrum of host immune responses. These parasites are transmitted to humans and other vertebrate hosts by the bite of an infected female phlebotomine sand fly, a tiny (2–3 mm long) vector insect. There are three main forms of human disease: cutaneous leishmaniasis (CL), visceral leishmaniasis (VL), also known as kala-azar, and mucocutaneous leishmaniasis (MCL). CL is the most common form, VL is the most severe form, and MCL is the most disabling form of the disease. These diseases have not only a wide variety of clinical manifestations, but also present great epidemiological diversity, occurring in more than 100 countries on five continents, where there is a total of 350 million people at risk and 12 million cases of infection. An estimated 300,000 new cases of VL and more than one million new cases of CL are reported annually [1,2].

Leishmaniasis, considered one of the most neglected tropical diseases, is also a veterinary problem. In fact, canine leishmaniasis (CanL) is not only a veterinary concern, with millions of dogs estimated to be infected in Europe, but also a public health issue due to the zoonotic potential of infection [3,4,5]. An increasing number of reports suggest that animal leishmaniasis is not restricted to dogs; it affects many other mammalian and even avian species. The impact of these potential reservoirs needs to be considered when developing control measures for zoonotic leishmaniasis [6].

In this Special Issue of *Microorganisms*, on Advances in *Leishmania* Research: From Basic Parasite Biology to Disease Control, we have invited original contributions in *Leishmania* research on basic parasite biology, drug and vaccine development, host–parasite interactions, epidemiology, and leishmaniasis diagnosis. A total of 72 authors contributed to 10 publications, comprising eight research articles and two reviews. The received contributions have provided relevant data on *Leishmania* biology, infection, and ecology.

Two manuscripts have provided new insights into *Leishmania* ecology of with updated evidence of infection in rodents [7] and Iberian lynxes [8].

Ghawar et al. reported natural *Leishmania* infections in 46 rodents of the genus *Jaculus* from Tataouine governorate, where leishmaniasis is endemic, in southern Tunisia [7]. Parasites were observed in 19 (41.3%) smears, while parasitic DNA was detected in 28 (60.9%) spleen samples. Among the latter, 16 (66.7%) were from 24 *Jaculus hirtipes* specimens and 12 (54.5%) were from 22 *Jaculus jaculus*. Internal transcribed spacer 1 (ITS-1) polymerase chain reaction (PCR) and subsequent restriction fragment length polymorphism revealed *Leishmania major* (52.2%) in 14 *J. hirtipes* and 10 *J. jaculus* specimens, *Leishmania killicki* (synonym with *Leishmania tropica*) was detected in two *J. hirtipes* specimens (4.3%). This finding represents the first evidence of natural infection with *Leishmania* parasites in rodents belonging to the genus *Jaculus,* and provides the rationale to consider them as potential reservoir hosts of *Leishmania* spp. in Tunisia and other parts of North Africa.

Lima et al. described the occurrence of *Leishmania infantum* infection among a population of 41 wild-born Iberian lynxes (*Lynx pardinus*) living in the Guadiana river valley in southern Portugal [8]. Eight out of thirty-five blood samples were positive for kinetoplast DNA, and ITS-1 sequencing confirmed *L. infantum* in two. Four and eight out of thirty-six lynxes were found to be seropositive by immunofluorescence antibody test (IFAT) and enzyme-linked immunosorbent assay (ELISA), respectively. Agreement between PCR, IFAT, and three ELISA antigens was found for one out of twenty-seven samples. Results suggest the susceptibility of autochthonous *L. pardinus* to *L. infantum*. Further investigation is required to assess the impact of *Leshmania* infections on the conservation of this feline species.

The special edition has also provided new advances for a better understanding of host-*Leishmania* interactions with Picard et al., describing new approaches for a better comprehension of infection [9]. The work showcased by Picard et al. presented the NoNo flow-cytometric approach as a possible new tool for understanding the interactions between *Leishmania* and the host [9]. With fluorescent parasites, these authors demonstrated that among primary human monocyte populations in whole blood, *L. infantum* preferentially infects classical (CD14+ CD16−) and intermediate (CD14+ CD16+) populations. Furthermore, although a subset of polymorphonuclear neutrophils are infected early in whole blood, they are less infected than monocytes.

Martí-Carreras et al., García-Hernández et al., and Silveira et al. in distinct publications used omics approaches for an enhanced understanding of parasite biology and phylogeny, with a focus on the problematic of resistance and treatment failure [10,11,12].

Martí-Carreras et al. [10], presented a genomic analysis platform (LeishGenApp) associated with Nanopore sequencing to demonstrate the capacity in four *L. infantum* strains to detect biologically relevant aspects such as copy number variations and genetic pharmacoresistance profiles. Overall, the conjugation of nanopore sequencing and the LEishGenApp was proposed as a tool to streamline biomarker research in *L. infantum.*

García-Hernández et al. [11] evaluated transcriptomic changes in *L. infantum* intracellular amastigotes isolated from human patients with leishmaniasis associated with treatment failure in infected THP-1 cells. The gene that codes for the prostaglandin f2α synthase seems to play an important role in pathogenicity and treatment failure, since it appeared upregulated in all of the *L. infantum* lines studied. Parasite adaptation leads to alterations in genes involved mainly in transport through cell membranes, energy and redox metabolism, and detoxification. Results showed that at a late timepoint post-infection, the transcriptome of *Leishmania* undergoes considerable changes that potentially improve the survival of the parasite lines in host cells, as well as to treatment failure.

Silveira et al. used comparative genomics to address the ancestry of strains belonging to the *Leishmania donovani* complex [12]. This is a controversial subject. The authors provided genomic evidence that supports the notion that *Leishmania chagasi*/*L. infantum chagasi* could be native to South America. Moreover, they propose the definition of a new *Leishmania* spp. associated with atypical dermal leishmaniasis in Central America.

The control of leishmaniasis is challenging. In this sense, vaccine development and improvement of the available techniques to detect infections are important challenges that were addressed in two different publications in the Special Issue [13,14]

The work by Fernández et al. examines the efficacy of a novel vaccine consisting of the *Leishmania* membrane protein KMP11, LEISH-F3+, and the sand fly salivary protein LJL143 [13]. The responses were greater when higher doses of KMP11 and LEISH-F3+ proteins were administered along with the GLA-SE adjuvant and/or when delivered within virosomes (VS). Hamsters immunized with the complete combination (i.e., all antigens in vs. + GLA-SE) showed significantly lower parasite burdens in the spleen compared to controls. Results indicate that this innovative vaccine formulation confers protection against *L. infantum* infection, supporting the advancement of the vaccine formulation towards future phase I human clinical trials.

Employing ELISA, Lima, C.S. et al. evaluated six *Leishmania*-specific antigens for detecting infection in a cohort of 390 from geographical areas where CanL is endemic in Portugal [14]. Seroprevalence ranged from 15.4% to 23.1%, depending on the antigen. Only 8.2% of the dogs were seropositive for all the *Leishmania* antigens and a further 31.0% had antigen-dependent seropositivity. A serological score was validated and revealed a general positivity of 26.9%. This work highlighted the limitation of single-antigen serological surveys. The establishment of serological profiles might be used not only to identify infections but also to address vaccination status and treatment efficacy.

The special edition on Advances in *Leishmania* Research: From Basic Parasite Biology to Disease Control also contains two reviews that address relevant subjects such as the impact of host–parasite interactions and parasite–parasite interactions in parasite biology and infectivity [15], and also a second review that introduces the concept of vesicle depleted exoproteome (VDE) [15,16].

The review by das Chagas et al. focused on the surprising adaptative capabilities of *Leishmania* protozoa, which go beyond genetic diversity to include variability between parasitic cells during infection of their hosts [15]. According to these authors, a single cell might shelter the intrastrain variability required to overcome disparate milieus, to adapt and thus survive and propagate. Strains and species living in the same environment can also compete apart from cooperating. The parasite’s genetic background is a source of phenotypic variability, but host immunity may also represent an important determinant of challenge. The complex balance of multiple factors and features, including multiple infections, can lead to different disease outcomes and prognoses.

The review by Esteves et al. provided an overview of the exoproteome of *Leishmania* spp., introducing the concept of VDE [16]. This review provides for the first time a systematic view of the relevance of studying the VDE and the possible impact of these studies not only for a better understanding of parasite biology, infectivity, and disease, but also as a relevant source of antigens for diagnostic and vaccine development.

Overall, the main goal of this Special Issue has been accomplished, providing new and exciting current research on *Leishmania* parasites and the diseases they cause and strongly suggesting that control must be an integrated approach, whether in humans or in other animals, as an example of One Health approach to leishmaniasis. We hope you will enjoy reading this selection of papers.
